# Studies on the Anticancer and Antioxidant Activities of Resveratrol and Long-Chain Fatty Acid Esters

**DOI:** 10.3390/ijms24087167

**Published:** 2023-04-12

**Authors:** Patrycja Szczepańska, Magdalena Rychlicka, Sylwia Groborz, Angelika Kruszyńska, Rodrigo Ledesma-Amaro, Andrzej Rapak, Anna Gliszczyńska, Zbigniew Lazar

**Affiliations:** 1 Department of Biotechnology and Food Microbiology, Wroclaw University of Environmental and Life Sciences, Chelmonskiego 37, 51-630 Wroclaw, Poland; 2 Department of Food Chemistry and Biocatalysis, Wroclaw University of Environmental and Life Sciences, Norwida 25, 50-375 Wroclaw, Poland; 3 Department of Experimental Biology, Wroclaw University of Environmental and Life Sciences, ul. Norwida 27B, 50-375 Wroclaw, Poland; 4 Hirszfeld Institute of Immunology and Experimental Therapy, Polish Academy of Sciences, R. Weigla 12, 53-114 Wroclaw, Poland; 5 Department of Bioengineering and Imperial College Centre for Synthetic Biology, Imperial College London, London SW7 2AZ, UK

**Keywords:** resveratrol esters, resveratrol, long-chain fatty acids, anticancer properties, antioxidant properties

## Abstract

Resveratrol (RES) is gaining recognition as a natural bioactive compound. To expand the possible applications of RES with its enhanced bioactivity as well as to increase the health benefits of long-chain fatty acids, a lipophilization process of RES was performed using three fatty acids: palmitic acid (PA), oleic acid (OA), and conjugated linoleic acid (CLA). The obtained mono-, di-, and tri-esters of RES were evaluated for their anticancer and antioxidant properties against lung carcinoma (A549), colorectal adenocarcinoma (HT29), and pancreatic ductal adenocarcinoma (BxPC3) cell lines. Human fibroblast (BJ) cells were used as a control. Several parameters were investigated: cell viability and apoptosis, including the expression of major pro- and anti-apoptotic markers, as well as the expression of superoxide dismutase, a key enzyme of the body’s antioxidant barrier. Three of the obtained esters: mono-RES-OA, mono-RES-CLA, and tri-RES-PA, which significantly reduced the tumor cell viability up to 23%, at concentrations 25, 10, 50 μg/mL, respectively, turned out to be particularly interesting. The above-mentioned resveratrol derivatives similarly increased the tumor cells’ apoptosis by modifying their caspase activity of pro-apoptotic pathways (p21, p53, and Bax). Moreover, among the mentioned esters, mono-RES-OA induced apoptosis of the analyzed cell lines most strongly, reducing the number of viable cells up to 48% for HT29 cells versus 36% for pure RES. Furthermore, the selected esters exhibited antioxidant properties towards the normal BJ cell line by regulating the expression of major pro-antioxidant genes (superoxide dismutases—*SOD1* and *SOD2*) without the effect on their expression in the tumor, and therefore reducing the defense of cancer cells against increased oxidative stress induced by high ROS accumulation. The obtained results indicate that the esters of RES and long-chain fatty acids allow enhancement of their biological activity. The RES derivatives have the potential for being applied in cancer prevention and treatment, as well as for oxidative stress suppression.

## 1. Introduction

The synthesis of resveratrol derivatives and their novel enriched properties have been a hot research topic recently. Resveratrol (RES; 3,5,4′-trihydroxystilbene) is a naturally occurring polyphenolic stilbene, found mainly in the skin of grapes; however, it can also be found in other fruits such as blueberries, cranberries, rhubarb, passion fruit, and in their processed products [1]. RES belongs to the phytoalexin group of antibiotics and is produced by several plants in response to injury, infections, pathogens, and environmental stress [2]. The biological effects of RES are associated with human health benefits and have been extensively studied in vitro and in vivo [3]. Some of the described effects include its anti-inflammatory, antioxidant, antidiabetic, neuroprotective, cardioprotective, and anticancer properties [3]. Furthermore, a large number of preclinical studies indicate that RES is a promising drug for the prevention and treatment of cancer [4]. Nevertheless, RES, like other polyphenols, has a high oral absorption and rapid, extensive metabolism, which can be associated with poor bioavailability. As a result, only trace amounts of unchanged RES can be found in the systemic circulation, limiting its effective use in practical medicine [5]. Moreover, utilization of RES may be hindered by its hydrophilicity while being used in lipophilic systems [6]. Therefore, structural modification of RES may provide an opportunity to address the above-mentioned concerns. In fact, there are several available scientific studies on lipophilic derivatives of phenolic compounds. These studies were focused on the maximized antioxidant activities of polyphenols as well as their higher performance in biological model systems, especially via structure modification, such as those for epigallocatechin gallate (EGCG) [6] or rosmarinic acid [7]. Moreover, EGCG esterified with docosahexaenoic acid (DHA) provided a novel bioactive product being a composite of two bioactive compounds, since DHA itself possesses health benefits [8]. Likewise, RES esterified with fatty acid chlorides of different chain lengths (C3:0–C22:6) resulted in higher antioxidant activity for RC20:5n-3 (REPA) and RC22:6n-3 (RDHA) esters and better hydrogen peroxide scavenging activity (RC3:0–RC14:0) compared to pure RES [9]. The RES analogue 4,4′-dihydroxy-trans-stilbene, containing two hydroxyl groups in the *para*-positions 4 and 4′, showed higher antiproliferative activity than pure RES [10]. In mouse and zebrafish lung cancer models, RES derivatives significantly inhibited tumor volume, cell proliferation, and tumor angiogenesis and caused significantly reduced liver metastatic lesions. In similar experiments, RES with hydroxyl groups in positions 3′, 4, and 5′ showed even higher cytotoxicity in HL-60 cells compared to RES, indicating an increased antitumor activity [11]. Recent studies showed that chemically synthesized RE-butyric acid (RE-B) ester has a higher ability to decrease liver fat accumulation and antioxidant capacity than RES itself [9,12].

In the case of oral administration of ester compounds, the ester bond may be rapidly hydrolyzed. In 2007, Biasutto and coworkers reported that some quercetin esters are partially hydrolyzed, while passing through a monolayer of MDCK-1, MDCK-2, and Caco-2 cells. The hydrolysis of the ester was dependent on the type of compound which was examined [13]. According to Pokorski and colleagues (2003), ascorbyl palmitate-treated cats showed more recovery of ascorbate in their brain tissues compared to ascorbic acid-treated cats [14]. Therefore, it can be hypothesized that RES esters may exhibit beneficial effect even if hydrolyzed. 

In addition, lipophilic phenolic derivatives also offer several advantages over the original phenols, including improved pharmacological profiles and bioavailability. Polyphenols prevent fatty acid oxidation, assisting their intestinal uptake, and preserve their bioactivity [15], while fatty acids influence the metabolism and bioaccessibility of polyphenols [16]. The latter, when conjugated with fatty acids (as lipophenols) show increased lipophilicity, cell penetration, as well as bioavailability of specific polar phenolic drugs, reaching appropriate solubility of hydrophobic drugs and can become tissue/tumor-specific [17,18]. 

Collectively, all the information gathered above suggests that lipophilic esters of RES and long-chain fatty acids may represent novel drug candidates with improved anticancer and antioxidant properties; however, their mechanism of action is not yet fully understood and requires further research. In this study, RES was esterified with selected fatty acids; palmitic acid (C16:0), oleic acid (C18:1), and conjugated linoleic acid (C18:2) and mono-, di-, and tri-esters were obtained. The synthesized lipophilic RES derivatives were then tested to identify and evaluate their effect on viability, apoptosis, and antioxidant properties on various cancer lines. 

## 2. Results and Discussion

### 2.1. Preparation of Esters of Resveratrol and Fatty Acids

**Figure 1:**
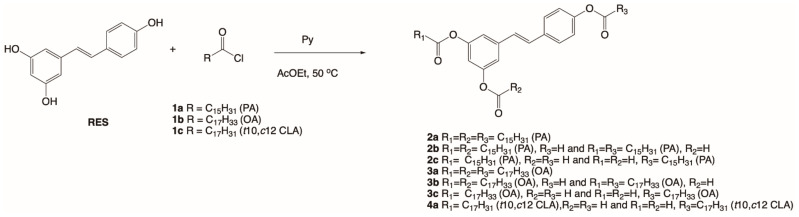
Scheme of chemical acylation of resveratrol.

In the presented work, the lipophilization of RES to improve its therapeutic potential as an anticancer agent was performed. RES is known for its cancer chemo-protective activity. It acts on three major stages in carcinogenesis, namely anti-initiation, anti-promotion, and anti-progression [19]. It was hypothesized that the increase in lipophilicity of this natural stilbenoid will allow for obtaining derivatives with higher antiproliferative and antioxidant activity. Therefore, we carried out the process of lipophilization using selected fatty acids: palmitic acid (PA) (16:0), oleic acid (OA) (18:1), and conjugated (10*E*,12*Z*)-linoleic acid (CLA) (18:2). For this purpose, chemical acylation of hydroxy groups at 3, 5, and 4′ carbon atoms of RES using chlorides of PA, OA, and CLA was performed. The acyl groups reacted with different numbers of hydroxy groups located in different positions of RES depending on the nature of the electrophile and the reaction conditions. After 3 h of reaction mixtures of mono-, di-, and tri-esters of RES, palmitic or oleic acid were obtained, whereas the reaction of RES with CLA chloride resulted in only mono-*O*-(conjugated)linoleoylresveratrol (Figure 1). The obtained products were separated and purified by column chromatography and their structures were determined using spectroscopic analysis and compared to the literature data. The partial esterification of the above-mentioned groups as well as incorporation of long-chain saturated or unsaturated fatty acids into RES allowed us to examine the structure–activity relationship.

### 2.2. RES Esters Inhibit Growth of Various Cancer Cell Lines

**Figure 2:**
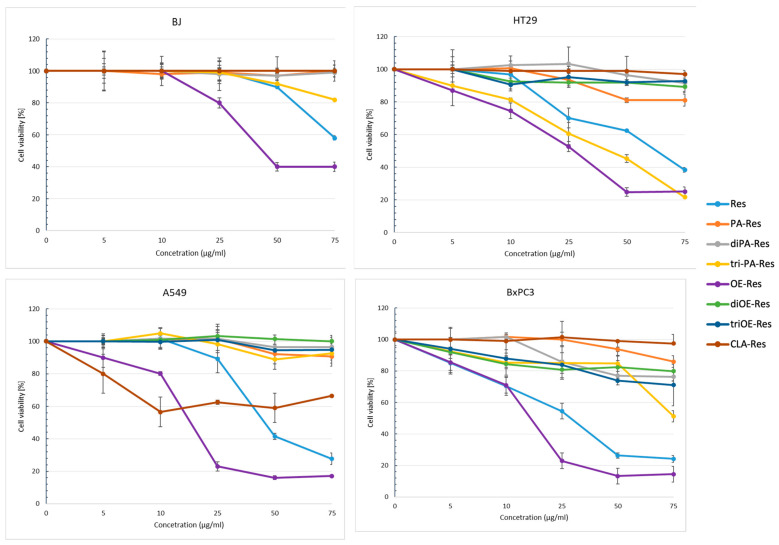
Effects of LCFAs-RES esters on cancer cell viability. A549, BxPC3, HT29, and BJ cells were treated with RES derivatives at different concentration (5–75 µg/mL). Cell viability was assessed by MTS assay after 48 h exposure. Results are expressed as mean ± SE of three separate experiments.

We evaluated the effect of all LCFAs-RES esters on the viability of three cancer cell lines, lung adenocarcinoma cell line A549, pancreatic ductal adenocarcinoma cell line BxPC3, and colorectal cancer cell line HT29, and normal cell line BJ as a control. We examined the effects of these compounds on the growth of cells using MTS assay. Cell lines used in these studies were chosen due to the high incidence of these cancers, their high resistance to chemotherapy, and difficulties in their treatment. The cells were treated for 48 h with a wide range of concentrations of RES derivatives ranging from 5 to 75 µg/mL for each ester. The cytotoxicity results are presented in Figure 2. 

We observed mono-RES-OA (**3c**), tri-RES-PA (**2a**), and mono-RES-CLA (**4a**) esters exhibiting the highest cell viability reduction. The mono-RES-OA acted on all cancer lines better than RES, causing inhibition of cell viability starting from the lowest dose of 5 µg/mL. Similar results were observed for tri-RES-PA in the case of HT29 cells and for mono-RES-CLA acting on the A549 cell line. Moreover, most of these compounds did not inhibit the viability of the human normal BJ cells, except the mono-RES-OA, which showed a strong reduction in their viability at concentration higher than 25 µg/mL.

Significant inhibition was observed for cells treated with mono-RES-OA at a concentration of 25 µg/mL, equal to 23% for the A549 and BxPC3 cells and 53% for HT29 cells. This cell viability reduction was significantly higher compared to their treatment with RES at the same concentration, reaching 90% for A549, 55% for BxPC3, and 70% for HT29 lines, respectively. For HT29 cells, strong viability inhibition was also shown by tri-RES-PA ester reaching 25% of cell viability when cells were treated with 50 µg/mL, while the inhibition reached by the RES treatment was about 2 times lower. In turn, mono-RES-CLA ester reduced cell viability to 55% for A549 cells at a concentration of 10 µg/mL, whereas no effect on these cells treated with 10 µg/mL RES was observed. Interestingly, the rest of the esters had no significant effect on cancer cell viability. 

Several studies have demonstrated anticancer activity of RES and its derivatives. Won Young Oh et al. [20] investigated the anticancer activity of RES and its mono-esters, resveratryl propionate (RC3:0) and resveratryl docosahexaenate (RDHA), using liver cancer (HepG2), colon cancer (HT-29, A431), breast cancer (MCF7), and gastric cancer (AGS) cell lines. According to their results, RES with RC3:0 at a concentration of 50–75 µg/mL reduced cell viability of most lines below 40%; in contrast, RDHA showed the lowest cytotoxicity at all concentrations. Zhu et al. [21] investigated the antiproliferative properties of a series of RES-based aspirin prodrugs. The authors synthesized RSV–aspirin hybrid (RAH), by esterification of the 4′-phenolic hydroxyl of RES with aspirin (acetylsalicylic acid), also obtaining its regioisomer, formed by esterification of the 3-phenol hydroxyl of RES. The anticancer properties of the two compounds were evaluated in human colorectal cancer cells HCT-116 and HT-29 using the MTT assay. As a result, both compounds inhibited the growth of cancer cells in a dose-dependent manner. The observed effect was superior to that demonstrated by administration of RES and acetylsalicylic acid alone or simultaneously. Notably, RAH turned out to be more efficient than its regioisomer, suggesting that the position of esterification influences antitumor activity. Similar results were obtained in our study, confirming that the two free hydroxyl groups at C-3 and C-5 in ring A of the RES moiety play an important role for its biological activity. Moreover, the esterification of RES with OA, PA, and CLA at the C-4 position also favors the anticancer activity of the corresponding esters. More recently, Peterson et al. [22] investigated the effect of ester-derived 4′-RSV on calcium dynamics in triple-negative breast cancer (TNBC), a highly aggressive subtype of breast cancer. Compounds were obtained by esterification of the 4′-hydroxy function of RSV with a pivalate, isobutyrate, or butyrate group, respectively. These compounds were investigated for their ability to reduce cell viability in MDA-MB-231 cancer cell lines using the MTT assay. As a result, both compounds, RSV 4′-pivalate and RSV 4′-isobutyrate, were more active than RES, reducing the cell viability to 14.14% and 7.70% vs. 58.45%, respectively. 

### 2.3. Apoptosis

**Figure 3:**
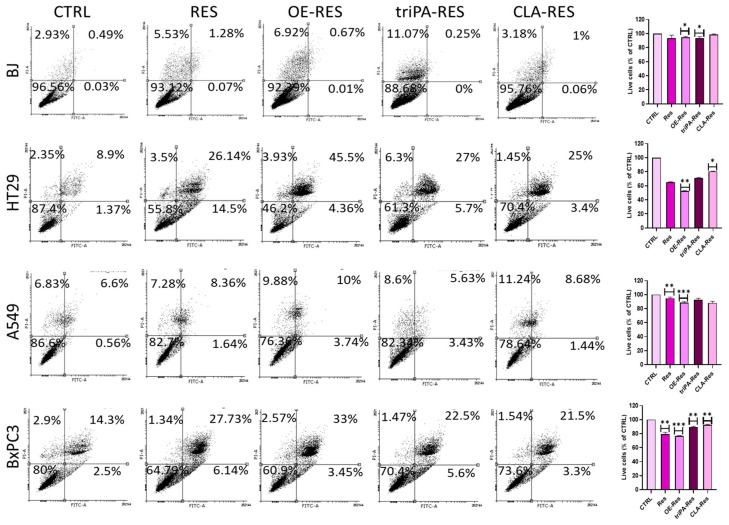
Induction of apoptosis in human cancer cells, HT29, A549, and BxPC3, after treatment with RES derivatives. Normal BJ cells were used as a control line. Representative FACS dot plots showing the effect of treatment with RES and mono-RES-OA (10 µg/mL) tri-RES-PA (50 µg/mL) and mono-RES-CLA (25 µg/mL) on phosphatidylserine exposure and plasma membrane integrity after 48 h of incubation with the cells, as determined by annexin V-FITC/PI staining. A comparison of treatment groups and untreated cells is shown by an asterisk (*). * *p* < 0.05, ** *p* < 0.01, *** *p* < 0.001.

**Figure 4:**
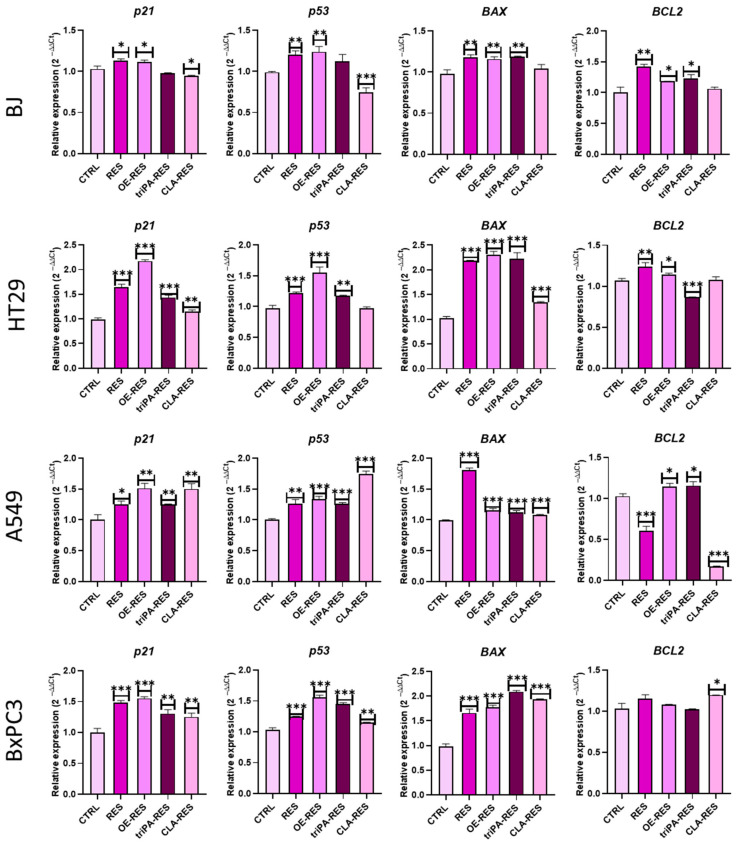
Expression of pro- and anti-apoptotic genes after treatment with RES esters in normal and cancer cells. Bar charts illustrating the relative expression of major apoptotic markers: *p21, p53, BAX,* and *BCL2* genes. A comparison of treatment groups and untreated cells is shown by an asterisk (*). * *p* < 0.05, ** *p* < 0.01, *** *p* < 0.001.

In the next step, the apoptosis level in A549, BxPC3, HT29, and BJ cell lines was analyzed (Figure 3) to verify if the inhibition of cell growth demonstrated in the MTS assay was related to apoptosis. The induction of apoptosis was investigated using Annexin V-FITC/PI double staining as well as by measuring gene expression of apoptosis master regulators (Figure 4). All tested cell lines were treated with different concentrations of every RES ester (RES, mono-RES-OA—10 µg/mL, tri-RES-PA—50 µg/mL, and mono-RES-CLA—25 µg/mL). The most appropriate concentrations were chosen after the MTS test, where the significant effect on cell viability was noted.

For the BJ line, no effect on apoptosis was noted after treatment with RES esters, which is consistent with the results obtained in the MTS test. These results proved that RES derivatives at the analyzed concentrations have no impact on the normal cell line. In contrast, HT29 and BxPC3 cancer cells were prone to highly increased apoptosis after treatment with RES esters, as evidenced by the decrease in the number of living cells and the consequent increase in the number of apoptotic cells (Figure 3). As in the MTS test, the mono-RES-OA most significantly induced apoptosis in both cell lines, reducing the number of live cells by 48% and 31%, respectively. This result confirms the stronger cytotoxic effect of this ester against the investigated cancer cell lines compared to pure RES (36% in HT29, 27% in BxPC3). The apoptotic effect in both lines was also visible in samples treated with tri-RES-PA, where the number of live cells was reduced to 61.3% for HT29 and to 70.4% for BxPC3, and for cells treated with mono-RES-CLA to 70.4% (HT29) and 73.6% (BxPC3), respectively. The A549 cell line was the most resistant, where we expected increased apoptosis when cells were treated with the mono-RES-CLA; however, only a slight decrease (less than 10%) in the number of live cells was recorded. A similar effect was shown for mono-RES-OA. 

Similarly, the relative expression of pro- and anti-apoptotic markers was assessed at the mRNA level using the RT-qPCR approach. The obtained data showed that treated HT29 and BxPC3 cancer cells were characterized by a significant upregulation of key pro-apoptotic factors, including *p21*, *p53,* and *Bax,* compared to the untreated cells (Figure 4). In contrast, the apoptosis inhibitor *BCL-2* showed no significant difference in expression in either HT29 or BxPC3 cells. Surprisingly, increased expression of pro-apoptotic genes was also observed in the A549 cells, especially during mono-RES-CLA treatment, which was accompanied by downregulation of the *BCL-2* gene. In the normal BJ cell line, the expression of investigated genes showed no significant changes.

The results suggest that the mono-RES-OA ester showed the strongest cytotoxic activity against the investigated tumor cell lines. Treatment of HT29 and BxPC3 cells with mono-RES-OA enabled a significant increase in apoptosis by enhancing the overexpression of *p21, p53,* and *Bax*, suggesting that mono-RES-OA has a very strong pro-apoptotic effect without simultaneously affecting human normal cells. 

### 2.4. Antioxidant Activity

**Figure 5:**
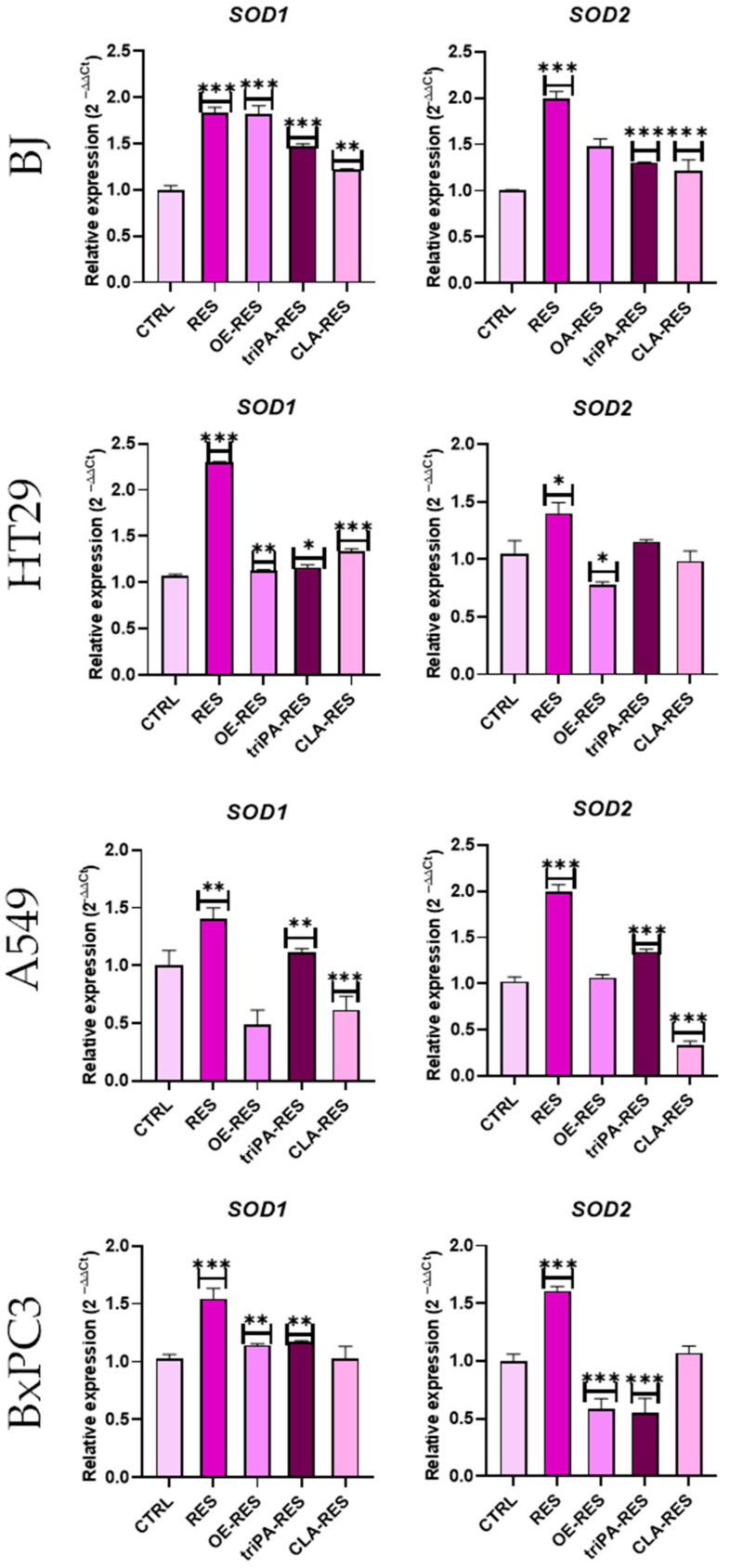
Effect of RES esters on oxidative stress in the normal and cancer cells. Relative gene expression of *SOD1* and *SOD2* transcripts. A comparison of treatment groups and untreated cells is shown by an asterisk (*). * *p* < 0.05, ** *p* < 0.01, *** *p* < 0.001.

The activity of superoxide dismutase (*SOD*) is an important indicator of intracellular antioxidant power, which converts superoxide anions into H_2_O_2_ and O_2_ [23]. The most common *SOD* isomerases are the cytoplasm-localized copper–zinc SOD1 (CuZnSOD) and the mitochondria-localized manganese SOD2 (MnSOD). In this study, the preliminary assessment of the antioxidant activity measured by the expression of the *SOD1* and *SOD2* genes was carried out using RT-qPCR (Figure 5). The results showed that all of the compounds caused a significant increase in the expression level of *SOD1* and *SOD2* genes on the healthy BJ cells, confirming the antioxidant properties of the tested compounds. This result is consistent with previous studies, where the properties of the esterified RES in food were investigated [9]. According to that study, RES derivatives showed better antioxidant activity compared to RES alone in a bulk oil system. The RES esters RC20:5n-3 and RC22:6n-3 showed the highest antioxidant activity when added to ground meat. Moreover, they inhibited copper-induced LDL oxidation and hydroxyl radical-induced DNA breakdown. In another study by Ming-Kuei Shih and colleagues [24], authors reported higher oxidative capacity of RES derivatives as well as indicated that their activity is related to the number and position of butyrate esterification sites. In our study, in the case of cancer cells, RES esters had either no or a slight effect on the expression of *SOD* genes. A significant dysregulation of the *SOD1* gene expression was noted on the A549 line with mono-RES-OA treatment and of the *SOD2* gene on the BxPC3 line with tri-RES-PA treatment. In contrast, tumor cells treated with RES showed increased expression of both genes.

The activity of *SOD1* and *SOD2* genes in cancer cells often depends on the stage of the tumor, as well as its cellular location [25]. Tumor cells contain higher levels of reactive oxygen species (ROS) compared to normal cells, mostly due to their accelerated metabolism, which is required to maintain their high proliferation rate. Due to the formation of excessive amounts of ROS characteristic of tumorigenic processes, most cancer cells show increased overexpression of *SOD*. Furthermore, there are many studies reporting that RES and its derivatives, as well as fatty acids themselves, cause increased ROS accumulation in cancer cells [15,24,26,27,28]. 

The obtained results show that RES esters had no effect on the expression of the main pro-antioxidant genes in cancer cells, decreasing the chances of defense of these cells against the increased oxidative stress induced by high ROS accumulation. In contrast, the elevated SOD expression caused by treatment with pure RES can be explained by the fact that the compound has a negative effect on the cells. Cells, protecting themselves, overexpress SOD (disruption of homeostasis) genes; however, the expression level is not enough to prevent apoptosis.

## 3. Materials and Methods

### 3.1. Materials

Substrates and solvents were of reagent grade and were used without further purification. Resveratrol was a gift from Hansen Supplements. Conjugated (10*E*,12*Z*)-linoleic acid (CLA), acyl chlorides (palmitoyl chloride and oleoyl chloride), pyridine, propidium iodide (PI), penicillin, streptomycin, and neomycin were obtained from Sigma-Aldrich (St. Louis, MO, USA). All organic solvents were purchased from Merck. RPMI 1640, DMEM, and Fetal Bovine Serum (FBS) were obtained from Thermofisher Scientific (Grand Island, NY, USA).

### 3.2. Methods of Analysis

Thin layer chromatography (TLC) analysis was carried out on a 0.2 mm pre-coated aluminum sheet 60 F_254_ plate (Merck Ltd., Darmstadt, Germany) with the mixture of hexane/ethyl acetate/formic acid (80:20:2, *v*/*v*/*v*). The compounds were detected using a solution of 10 g of Ce(SO_4_)_2_ and 20 g of phosphomolibdenic acid in 1 L of 10% H_2_SO_4_ followed by heating. The products were purified using column chromatography (CC) on silica gel (230–400 mesh). The solvent ratio was gradients of hexane/ether (2:1 next 9:1), hexane/ethyl acetate/formic acid (30:1:1.2 then 30:20:1). Each fraction was collected and monitored by TLC. Each pure product was procured following solvent removal. Spectroscopic analysis in the range of Nuclear Magnetic Resonance (^1^H NMR, ^13^C NMR, COSY, HSQC) was recorded on a Bruker Advance II 600 MHz spectrometer (Bruker, Rheinstetten, Germany). Samples were dissolved in CDCl_3_ and the chemical shifts of detected signals were referenced to the signals of residual solvent (δH = 7.26, δC = 77.00). 

### 3.3. Synthesis of CLA Chloride 

Conjugated linoleic acid (400 mg, 1.43 mmol) was dissolved in anhydrous methylene chloride solution (10 mL) and oxalyl chloride (1.23 mL, 14.1 mmol) was added dropwise to the mixture. Next the reaction mixture was stirred at room temperature. After 1 h the reaction was stopped. Excess oxalyl chloride and the solvent were evaporated under reduced pressure to make the product, which was next used for synthesis without purification. 

### 3.4. Synthesis of Lipid Derivatives of Resveratrol (2a-c-3a)

Resveratrol (200 mg, 0.876 mmol) was dissolved in ethyl acetate (20 mL) and then pyridine (300 μL, 3.702 mmol) was added. Esterification of resveratrol was carried out with acyl chlorides (PA-Cl, OA-Cl, CLA-Cl) at a mole ratio of 1:1. They were added dropwise to a solution of resveratrol in ethyl acetate. The reaction proceeded for 3 h under reflux and a blanket of nitrogen at 50 °C. Upon completion of the reaction, the mixture was allowed to stand until it reached room temperature and was washed 3 times with distilled water (60 °C). The ethyl acetate layer was then passed through a layer of anhydrous sodium sulfate, followed by the removal of the solvent. Crude products were purified by column chromatography on silica gel, as described in Section 2. The yields and the spectroscopic data of the products are given below and were confirmed by comparing the chemical shifts of resveratrol and its derivatives as well as with the literature data [9]. 

#### 3.4.1. Tri-O-palmitoylresveratrol (tri-RES-PA) (**2a**)

Colourless solid (60 mg, 9% yield, R_f_ = 0.9); 

^1^H NMR (600 MHz, CDCl_3_/CD_3_OD 2:1 (*v*/*v*), δ: 0.81 (t, *J* = 6.7 Hz, 9H, CH_3_(CH_2_)_13_CH_2_C(O)), 1.19–132 (m, 72H, CH_3_(CH_2_)_12_CH_2_CH_2_C(O)), 1.68 (m, 6H, CH_3_(CH_2_)_12_CH_2_CH_2_C(O)), 2.48 (t, *J* = 7.5 Hz, CH_3_(CH_2_)_12_CH_2_CH_2_C(O)), 6.73 (m, 1H, H-4), 6.90 (d, *J* = 16.0 Hz, 1H, H-7), 6.98 (d, *J* = 16.0 Hz, 1H, H-8), 7.01 (m, 2H, H-3′and H-5′), 7.03 (m, 2H, H-2 and H-6), 7.30 (m, 2H, H-2′ and H-6′).

#### 3.4.2. Di-O-palmitoylresveratrol (di-RES-PA) (**2b**)

Colourless solid (148 mg, 28% yield, R_f_ = 0.5); 

^1^H NMR (600 MHz, CDCl_3_/CD_3_OD 2:1 (*v*/*v*), δ: 0.81 (t, *J* = 7.0 Hz, 6H, CH_3_(CH_2_)_13_CH_2_C(O)), 1.19–1.32 (m, 48H, CH_3_(CH_2_)_12_CH_2_CH_2_C(O)), 1.56 (m, 4H, CH_3_(CH_2_)_12_CH_2_CH_2_C(O)), 2.28 (t, *J* = 7.5 Hz, CH_3_(CH_2_)_12_CH_2_CH_2_C(O)), 6.53 (m, 1H, H-4), 6.61 (d, *J* = 16.0 Hz, 1H, H-7), 6.87 (d, *J* = 16.0 Hz, 1H, H-8), 6.61–6.87 (m, 4H, H-3′, H-5′, H-2 and H-6), 7.15–7.29 (m, 2H, H-2′ and H-6′).

#### 3.4.3. Mono-O-palmitoylresveratrol (mono-RES-PA) (**2c**)

(132 mg, 37% yield, R_f_ = 0.2); 

^1^H NMR (600 MHz, CDCl_3_/CD_3_OD 2:1 (*v*/*v*), δ: 0.76 (t, *J* = 6.8 Hz, 3H, CH_3_(CH_2_)_13_CH_2_C(O)), 1.15–1.31 (m, 24H, CH_3_(CH_2_)_12_CH_2_CH_2_C(O)), 1.64 (m, 2H, CH_3_(CH_2_)_12_CH_2_CH_2_C(O)), 1.93 (t, *J* = 6.8 Hz, CH_3_(CH_2_)_12_CH_2_CH_2_C(O)), 6.31 (m, 1H, H-4), 6.81 (d, *J* = 16.2 Hz, 1H, H-7), 6.87 (d, *J* = 16.2 Hz, 1H, H-8), 6.69-6.98 (m, 4H, H-3′, H-5′, H-2 and H-6), 7.23–7.39 (m, 2H, H-2′ and H-6′).

#### 3.4.4. Tri-O-oleoylresveratrol (tri-RES-OA) (**3a**)

(32 mg, 3.6% yield, R_f_ = 0.88); 

^1^H NMR (600 MHz, CDCl_3_/CD_3_OD 2:1 (*v*/*v*), δ: 0.88 (t, *J* = 7.0 Hz, 9H, CH_3_(CH_2_)_7_CHCH (CH_2_)_7_C(O)), 1.26 (m, 60H, CH_3_(CH_2_)_7_CHCH (CH_2_)_7_C(O)), 1.74–1.76 (m, 6H, CH_2_-3″), 2.01–2.03 (m, 12H, CH_2_-8″ and CH_2_-11″), 2.55 (t, *J* = 7.5 Hz, 6H, CH_2_-2″), 5.35–5.36 (m, 2H, H-9″ and H-10″), 6.80 (m, 1H, H-4), 6.97 (d, *J* = 16.0 Hz, 1H, H-7), 7.05 (d, *J* = 16.0 Hz, 1H, H-8), 7.07 (m, 2H, H-3′and H-5′), 7.10 (m, 2H, H-2 and H-6), 7.48 (m, 2H, H-2′ and H-6′).

#### 3.4.5. Di-O-oleoylresveratrol (di-RES-OA) (**3b**)

(188 mg, 28% yield, R_f_ = 0.5); 

^1^H NMR (600 MHz, CDCl_3_/CD_3_OD 2:1 (*v*/*v*), δ: 0.88 (t, *J* = 6.8 Hz, 6H, CH_3_(CH_2_)_7_CHCH (CH_2_)_7_C(O)), 1.27 (m, 40H, CH_3_(CH_2_)_7_CHCH (CH_2_)_7_C(O)), 1.61–1.65 (m, 4H, CH_2_-3″), 2.00–2.04 (m, 8H, CH_2_-8″ and CH_2_-11″), 2.35 (t, *J* = 7.5 Hz, 4H, CH_2_-2″), 5.34–5.36 (m, 4H, H-9″ and H-10″), 6.49 (m, 1H, H-4), 6.79–6.80 (m, 2H, H-3′and H-5′), 6.84 (d, *J* = 16.0 Hz, 1H, H-7), 6.90 (d, *J* = 16.0 Hz, 1H, H-8), 6.98–7.07 (m, 2H, H-2 and H-6), 7.35 and 7.46 (2m, 2H, H-2′ and H-6′).

#### 3.4.6. Mono-O-oleoylresveratrol (mono-RES-OA) (**3c**)

(224 mg, 52% yield, R_f_ = 0.26); 

^1^H NMR (600 MHz, CDCl_3_/CD_3_OD 2:1 (*v*/*v*), δ: 0.88 (t, *J* = 7.0 Hz, 3H, CH_3_(CH_2_)_7_CHCH (CH_2_)_7_C(O)), 1.27 (m, 20H, CH_3_(CH_2_)_7_CHCH (CH_2_)_7_C(O)), 1.74–1.77 (m, 2H, CH_2_-3″), 2.01–2.03 (m, 4H, CH_2_-8″ and CH_2_-11″), 2.55 (t, *J* = 7.6 Hz, 2H, CH_2_-2″), 5.35–5.36 (m, 2H, H-9″ and H-10″), 6.46 (m, 1H, H-4), 6.77 (d, *J* = 16.0 Hz, 1H, H-7), 6.92 (d, *J* = 16.0 Hz, 1H, H-8), 6.74–6.78 (m, 2H, H-3′and H-5′), 7.03–7.21 (m, 2H, H-2 and H-6), 7.30 and 7.43 (2m, 2H, H-2′ and H-6′).

#### 3.4.7. Mono-O-(conjugated)linoleoylresveratrol (mono-RES-CLA) (**3a**)

(100 mg, 23% yield, R_f_ = 0.16); 

^1^H NMR (600 MHz, CDCl_3_/CD_3_OD 2:1 (*v*/*v*), δ: 0.79 (t, *J* = 7.0 Hz, 3H, CH_3_-18″), 1.17–1.24 (m, 16H, CH_2_-4-8 and CH_2_-15-17), 1.52–1.54 (m, 2H, CH_2_-3″), 1.77–2.08 (m, 4H, CH_2_-9″ and CH_2_-14″), 2.19 (t, *J* = 7.2 Hz, 2H, CH_2_-2″), 6.16 (d, *J* = 15.0 Hz, 1H, H-10″), 6.47 (m, 1H, H-13″), 6.70–6.76 (m, 5H, H-4, H-12″, H-11″, H-3′and H-5′), 6.84 (d, *J* = 16.0 Hz, 1H, H-7), 6.88 (d, *J* = 16.0 Hz, 1H, H-8), 7.26–7.32 (m, 4H, H-2, H-6, H-2′ and H-6′).

### 3.5. Cell Lines and Cell Culture 

The human cancer cell lines A549 (lung carcinoma) and HT29 (colorectal adenocarcinoma) were cultured in DMEM high-glucose culture medium with 10% fetal bovine serum (FBS) and Antibiotic-Antimycotic Solution, and BxPC3 (pancreatic ductal adenocarcinoma) was maintained in RPMI 1640 culture medium supplemented with 2 mM L-glutamine, 100 U/mL penicillin, 100 µg/mL streptomycin, and 10% FBS. As a control, the BJ (human fibroblasts) cell line was used, maintained in modified Eagle’s MEM medium with 10% FBS. All cell lines were cultured at 37 °C in a humidified atmosphere of 5% CO_2_. The cells were seeded at densities of 5 × 103 cells/0.1 mL (0.32 cm^2^) (cell viability assay) and 5 × 104 cells/0.5 mL (1.9 cm^2^) (flow cytometry). All cell lines were obtained from the collection of the Hirszfeld Institute of Immunology and Experimental Therapy, Polish Academy of Sciences, Wroclaw, Poland.

### 3.6. Determination of Cell Viability

Cell viability was determined using CellTiter-Glo^®^ One Solution Assay (Promega, Madison, WI, USA). For determination of cell viability, cells were seeded in a 96-well plate (NUNC, Roskilde, Denmark) at a density of 8 × 103 cells overnight. Next, all cells were treated with Rev or Rev-LCFAs esters at 10, 25, 50, and 75 μg/mL and incubated in 200 μL of the above culture medium for 48 h. Following the incubation, 20 μL of MTS solution was added to each well for 2 h; next, absorbance at 490 nm was recorded by a plate reader. Each treatment within a single experiment was performed in triplicate.

### 3.7. Analysis of Apoptosis

Apoptosis was assessed by Annexin V Apoptosis Detection Kit (Santa Cruz Biotechnology, Dallas, TX, USA) according to the manufacturer’s protocol. Briefly, the cells were stained with Annexin V-FITC (8 μg/mL) and PI (5 μg/mL) for 15 min at RT in the dark. In between steps, the cells were washed with cold PBS (with Ca^2+^ and Mg^2+^) containing 2.5% FBS. Data were acquired on a FACSCalibur flow cytometer (Becton Dickinson, Franklin Lakes, NJ, USA) and analyzed using Flowing Software 2.5.1 software (Perttu Terho, Turku, Finland). Apoptosis was quantified as a percentage of both Annexin V-positive and Annexin V/PI-double-positive cells.

### 3.8. RNA Extraction and Real-Time Reverse Transcription PCR (qRT-PCR)

Total RNA was extracted from each cell line using EXTRAzol reagent (Blirt, Gdańsk, Poland) according to the manufacturer’s instructions. The concentration of RNA, quality, as well as purity were measured using a nanospectrophotometer (BioTek, Winooski, VT, USA). Transcription of RNA into cDNA was prepared using the PrimeScript™ RT Reagent Kit with gDNA Eraser (TaKaRa, Gdańsk, Poland) by means of a T100 Thermal Cycler (Bio-Rad, Hercules, CA, USA) according to the manufacturer’s instructions. 

The gene expression levels were evaluated by real-time reverse transcription polymerase chain reaction (RT-qPCR) using SensiFAST SYBR Green Kit (Bioline, London, UK) in a CFX Connect™ Real-Time PCR Detection System (Bio-Rad). Briefly, 10 μL total volume of each reaction consisted of 5 μL of SensiFAST SYBR Master mix, 2.5 μL of targeted primer, and 2.5 μL of tested cDNA. The Real-Time PCR program was conducted as follows: 95 °C for 2 min, then 41 cycles at 95 °C for 15 s, annealing for 30 s in temperature specified for tested primers, and elongation at 72 °C for 15 s. The qPCR results were replicated in 3 independent experiments, and then the statistics were determined. Relative gene expression was normalized by the reference gene glyceraldehyde 3-phosphate dehydrogenase (GAPDH) using the 2^−ΔΔCT^ method. Primers used are shown in Table 1.

### 3.9. Statistical Analysis

The obtained results were analyzed by a one-way variance analysis (ANOVA) using GraphPad Software 8 (San Diego, CA, USA) and post hoc Tukey’s test. Statistically significant results were marked with an asterisk: *p* < 0.05 (*), *p* < 0.01 (**), and *p* < 0.001 (***). Results are presented as statistical mean SD from at least three independent experiments.

## 4. Conclusions

The presented work focused on the anticancer and antioxidant properties of RES esterified with selected fatty acids. To date, this is the first report presenting studies on the anticancer properties of lipophilic esters of RES. The obtained results enabled the selection of three esters, mono-RES-OA, tri-RES-PA, and mono-RES-CLA, which clearly showed efficient reduction in cancer cell viability compared to other esters without affecting the normal cells. In particular, the mono-RES-OA showed the highest antitumor properties, and its effect was stronger than that of pure RES at the same concentration. Furthermore, expression assays including the main pro-antioxidant *SOD* genes showed that the selected esters exhibited antioxidant properties towards the normal BJ cell line without a similar effect in cancer cells, thus reducing the chances of defending these cells against increased oxidative stress induced by high ROS accumulation. These results prove that hydrophobization of RES improves its biological activity. Despite numerous reports concerning the influence of fatty acids on the metabolism and bioavailability of polyphenols, it can be assumed that the obtained RES esters may exhibit the above-mentioned characteristics. RES modified with fatty acids has poorer solubility in water, but when administered orally, it should have higher stability and bioavailability in the intestines, especially being nanoencapsulated, which will also improve its pharmacological properties. However, in order to confirm these assumptions, in vivo studies using various animal models are needed. Further studies are also required to gain an in-depth understanding of the mechanisms of action of selected RES esters against specific cancer cell lines.

## Figures and Tables

**Table 1:** Sequences of primers used in qPCR.

Gene	Primer Sequence (5′–>3′)
BAX	F: CGAGTGGCAGCTGAGATGTT
	R: AAGGAAGTCCAGTGTCCAGC
BCL2	F: ATCGCCCTGTGGATGACTGAG
	R: CAGCCAGGAGAAATCAAACAGAGG
p21	F: TGCCGAAGTCAGTTCCTTGT
	R: GTTCTGACATGGCGCCTCC
p53	F: TTTCGACATAGCGTGGTGGT
	R: CTCAAAGCTGTTCCGTCCCA
SOD1	F: CATTCCATCATTGGCCGCAC
	R: GAGCGATCCCAATCACACCA
SOD2	F: GGACAAACCTGAGCCCCAAT
	R: TTGGACACCAGCCGATACAG

BAX: BCL-2-associated X protein; BCL-2: B-cell lymphoma 2; p21: cyclin-dependent kinase inhibitor 1A; p53: tumor suppressor p53; SOD1: Superoxide dismutase [Cu-Zn]; SOD2: Superoxide dismutase [Mn].

## Data Availability

Data sharing not applicable.
